# Evaluation of the Digital Support Tool Gro Health W8Buddy as Part of Tier 3 Weight Management Service: Observational Study

**DOI:** 10.2196/62661

**Published:** 2025-05-16

**Authors:** Petra Hanson, Farah Abdelhameed, Mohammed Sahir, Nick Parsons, Arjun Panesar, Michaela de la Fosse, Charlotte Summers, Amit Kaura, Harpal Randeva, Vinod Menon, Thomas M Barber

**Affiliations:** 1 Warwickshire Institute for the Study of Diabetes, Endocrinology and Metabolism University Hospitals Coventry and Warwickshire NHS Trust Coventry United Kingdom; 2 Warwick Medical School University of Warwick Coventry United Kingdom; 3 DDM Health Limited Coventry United Kingdom; 4 Hammersmith Hospital Cardiology Department Imperial College London London United Kingdom

**Keywords:** digital, weight management, innovation, service delivery, weight, obesity, chronic disease, intervention, digital health, mental health, tool, BMI, body mass index, United Kingdom, overweight, diabetes mellitus, primary care, healthy lifestyle, promotion, patient engagement

## Abstract

**Background:**

The escalating prevalence of obesity worldwide increases the risk of chronic diseases and diminishes life expectancy, with a growing economic burden necessitating urgent intervention. The existing tiered approach to weight management, particularly specialist tier 3 services, falls short of meeting the population’s needs. The emergence of digital health tools, while promising, remains underexplored in specialized National Health Service weight management services (WMSs).

**Objective:**

This service evaluation study assessed the use, effectiveness, and clinical impact of the W8Buddy digital support tool as part of the National Health Service WMS.

**Methods:**

W8Buddy, a personalized digital platform, provides a tailored weight management plan to empower individuals and was collaboratively developed with input from patients, the clinical team, and DDM Health. It launched at the University Hospitals Coventry and Warwickshire tier 3 WMS in 2022. All patients accessing University Hospitals Coventry and Warwickshire WMS were offered W8Buddy as part of standard care. Data were analyzed using independent samples *t* tests and Fisher exact tests for continuous and categorical outcomes, respectively. Multiple linear regression analysis explored associations between participant weight, engagement with W8Buddy, and time in the service.

**Results:**

Complete datasets for weights were available for 421 patients (220 W8Buddy group and 192 nonuser control group). W8Buddy users, predominantly female (n=185, 84.1%) and Caucasian, had a mean age of 43 years, while nonusers averaged 46 years (*P*=.02). Starting weights were comparable: 134 kg in the W8Buddy group and 130.2 kg in controls (*P*=.14); however, W8Buddy users had slightly higher starting BMI (49.6 vs 46.8 kg/m^2^, *P*=.08). A total of 33.5% (n=392) of patients activated W8Buddy and engaged with it. There was significant weight loss among W8Buddy users, with a 0.74 kg monthly loss compared to standard care (β=–.74, 95% CI –1.28 to –0.21; *P*=.007). The longer an individual stayed in this study and used W8Buddy, the more weight was lost. W8Buddy users with type 2 diabetes mellitus experienced a significant hemoglobin A_1c_ reduction (59.8 to 51.2 mmol/mol, *P*=.02) compared to nonusers with type 2 diabetes. W8Buddy users also showed significant improvement across the Satisfaction With Life Scale, the Karolinska Sleepiness Scale, and quality of life visual analog scale (*P*<.001) during follow-up.

**Conclusions:**

Participants engaging with W8Buddy as part of a digitally enabled tier 3 WMS demonstrated significant improvements in clinical and psychological outcomes, with weight changes statistically significant compared to those not engaging with the digital tool. Reduction in hemoglobin A_1c_ was present in both groups; however, statistical significance was only reached among those engaging with W8Buddy. These findings suggest digital tools can augment traditional services and promote patient empowerment. Future studies must provide long-term data to understand if the benefits from the digital tool are sustained.

## Introduction

In the United Kingdom, 25% of men and 26% of women live with obesity [1]. Overweight and obesity are more prevalent in people living in the most deprived areas compared to the least deprived areas (72% and 58%, respectively) [1]. Similarly, excess weight disproportionately affects people in the Black ethnic group (72% compared to 64% in people from the White ethnic group) [1]. People living with obesity are at increased risk of chronic diseases (eg, type 2 diabetes mellitus [T2DM], cancer, and mental illnesses) and have reduced life expectancy [2]. Treatment of obesity and associated ill health was estimated to cost the National Health Service (NHS) £6.5 billion (US $8.63 billion) in 2022, with the annual full cost of obesity in the United Kingdom being estimated at £58 billion (US $10.62 billion) [3]. Treatment and prevention of obesity have been identified as a priority both from an NHS and a wider economic perspective, with a clear focus from the Office for Life Sciences on obesity as one of its health care missions [4]. The public health and economic burden of obesity is considered a worldwide epidemic requiring urgent action.

A tiered approach to weight management exists in the United Kingdom. Tiers 1 and 2 are delivered in community and primary care and consist of healthy lifestyle promotion, advice, and include components of diet, physical activity, and behavior change. Tier 3 is often delivered in secondary care and represents a specialist weight management service (WMS), providing intensive management and comprising dietitians, specialist doctors, and psychologists [5]. People with BMIs over 40 kg/m^2^ or over 35 kg/m^2^ with obesity-related conditions (eg, diabetes, hypertension, or obstructive sleep apnea) are eligible for referral to tier 3 WMS [6]. However, the current tier 3 WMS provision does not meet population needs. Evidence shows that only 10% of people living with BMIs over 40 kg/m^2^ received referral to WMS, with marked regional variation [7]. Despite a capacity for 35,000 patients in NHS WMS [8], an estimated 4 million people are eligible, highlighting a significant supply-demand mismatch [9].

The use of digital health tools for management of chronic diseases, including obesity, is rapidly expanding [10]. Current evidence supports the acceptability of digital tools for management of obesity in the community [11], and there is evidence for the acceptability of such tools in NHS tier 3 WMS [12-14]. However, there are currently no studies assessing outcomes for a digital tool specifically created for a specialist NHS WMS that is fully incorporated into the patient pathway.

In September 2022, an innovative digital support tool, Gro Health W8Buddy, was launched in the specialist WMS at University Hospital Coventry and Warwickshire Trust (UHCW). W8Buddy is a bespoke version of the NHS-certified app “Gro Health.” Gro Health is regulated as a class I medical device (Medicines and Healthcare Products Regulatory Agency–regulated with a European conformity mark) and is the highest ORCHA (Organisation for the Review of Care and Health Applications)-rated app, certified by NHS Digital Technology Assessment Criteria. Gro Health is a precision digital health platform that helps to reduce body weight, improve glycemic control, and well-being among people living with obesity, overweight, or with T2DM [15,16]. The bespoke version of Gro Health was created with patients and staff in tier 3 WMS, ensuring this digital tool is fit for the needs of its patients. Creation of this bespoke digital support tool was supported by various programs, including Health Education England Topol Digital Fellowship and the NHS Clinical Entrepreneur scheme [17]. Gro Health W8Buddy has been endorsed by the National Institute for Health and Care Excellence Early Value Assessment for Digital Technologies in Specialist Weight Management in October 2023 [18].

The main aim of this study was to evaluate the use, effectiveness, and clinical impact of the W8Buddy digital support tool as part of the NHS specialist WMS and thus contribute to the generation of evidence for the use of digital tools in WMS, as needed by the National Institute for Health and Care Excellence appraisal. Our primary objective was to assess the uptake and engagement of patients with the W8Buddy. Our secondary objective was to assess the clinical effectiveness of W8Buddy via changes in weight, hemoglobin A_1c_ (HbA_1c_), and mental health outcomes.

## Methods

### Intervention

#### Overview

W8Buddy is the result of a 3-year collaboration between the digital health company DDM Health, specialist WMS clinicians at UHCW, and patients attending tier 3 WMS. This collaboration ensured that expert insights and user experiences were integrated into the tool’s development, fostering a comprehensive and user-centric platform tailored to the needs of individuals undergoing specialist weight management.

W8Buddy provides a structured, multicomponent weight management program designed to support behavior change and long-term health improvements. The platform includes the following.

#### Structured Education

A series of online, evidence-based modules covering topics such as nutrition, physical activity, behavioral psychology, and medication adherence. These modules are delivered in a stepwise manner, with progress unlocking subsequent content. The modules are found below.

#### Coaching

Users receive asynchronous coaching that is personalized to their goals, dietary and cultural preferences, and delivered by human coaches in the patient’s native language. Coaches engage with users via in-app messaging to reinforce behavioral strategies. Coaches have access to a dashboard that collates all patient information (demographics, health, and engagement in the app), which is used to tailor a personalized care plan that is delivered through the app. Health coaches are British Dietetic Association–accredited dietitians, Association for Nutrition–accredited nutritionists, or British Psychological Society–accredited psychologists with degree-level qualifications in health who complete NHS Clinical Safety and Safeguarding training, data security or privacy training or General Data Protection Regulation, accredited training in motivational interviewing, Making Every Contact Count, trauma-informed care, “PHE” (Public Health England), “Talk About Weight” guidance, Doncaster Council’s “Compassionate Approach,” and diversity or cultural competencies.

#### Meal and Exercise Logging

Users can track food intake, exercise sessions, and lifestyle behaviors through an integrated logging system. The platform supports manual entry, barcode scanning, artificial intelligence–based image recognition, and integration with wearable devices to facilitate real-time tracking.

#### Weight Tracking

Users can self-report weight measurements, which are recorded within the platform. This feature enables trend visualization and allows users to correlate weight changes with lifestyle behaviors. Given the established association between frequent weight tracking and improved weight loss outcomes, this component plays a key role in the intervention.

#### Clinician Dashboard (GroCARE)

Clinicians have real-time access to user-generated data, enabling them to monitor patient engagement and intervene when necessary. For example, clinicians can track which patients are completing education modules, logging meals, and tracking their weight. If a user is not engaging with the program or if there is a discrepancy between self-reported weight and clinic-measured weight, clinicians can provide targeted support through messages and automated reminders. This means that clinicians can assess who is accessing education modules or tracking food in the form of a food diary. Clinicians can also assess if the user-reported weight tracking corresponds to the weight recorded in the clinic.

**Figure 1:**
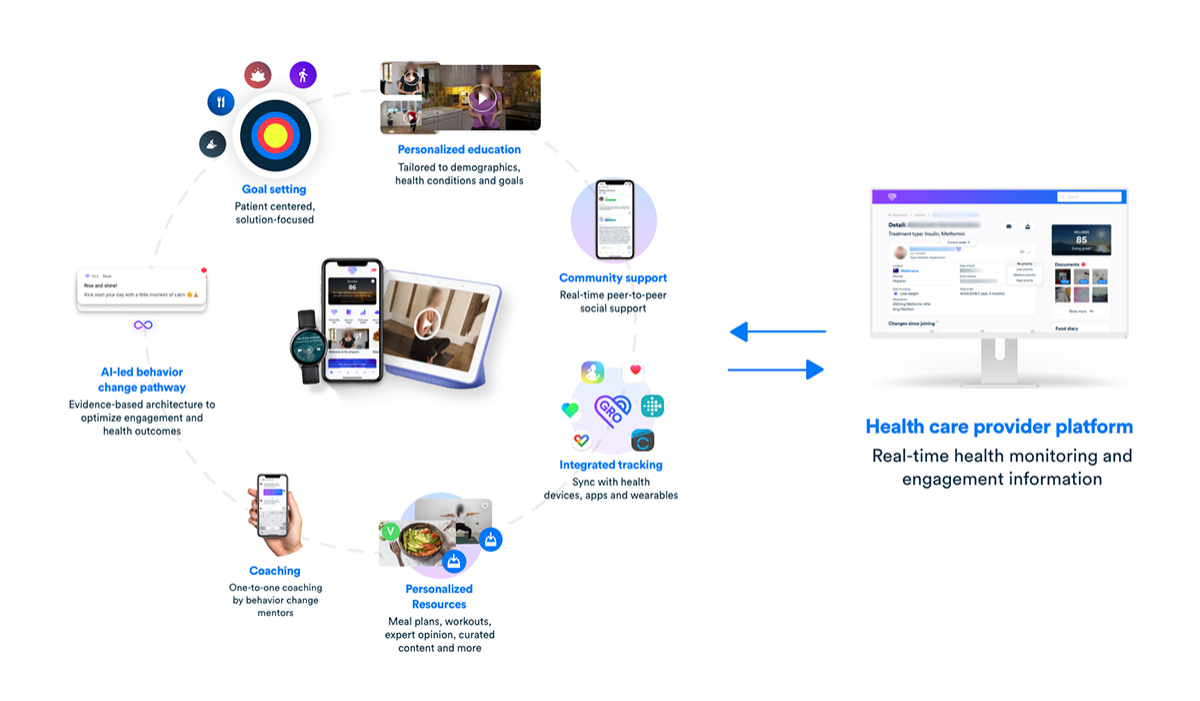
Overview of the features comprising Gro Health W8Buddy. AI: artificial intelligence.

Figure 1 provides an overview of the features comprising Gro Health W8Buddy.

W8Buddy is designed to be accessible across multiple platforms, including web-based (responsive) platforms, iOS, Android, wearable devices (smartwatches), smart televisions, and digital assistants such as Google Hub and Amazon Alexa, ensuring users can engage with the tool in a manner that suits their preferences and technological capabilities. The user experience is tailored to accommodate various factors, including individual goals, accessibility and literacy needs, language preferences, disease profiles, ethnicity, age, gender, and geographical location. This personalized approach aims to enhance the effectiveness of the intervention by catering to the unique needs and preferences of each user. The tool exhibits a strong commitment to inclusivity and representation, offering services in 23 languages and featuring culturally sensitive meal plans and resources, designed to enhance its acceptability and effectiveness among diverse populations, promoting a more inclusive approach to weight management.

The current standard care in tier 3 WMS includes assessment by an endocrinologist for medical conditions associated with obesity, specialist dietetic input, and in some cases, specialist psychological support. The exact number of appointments a patient receives is based on clinical need (eg, if new medication is started, more medical reviews are needed) and hence it is not possible to state the exact number of encounters with a health care professional. Patients who did not want to engage with W8Buddy received standard care, and patients who wanted to use W8Buddy were given access to W8Buddy in addition to standard care.

### Participants

**Figure 2:**
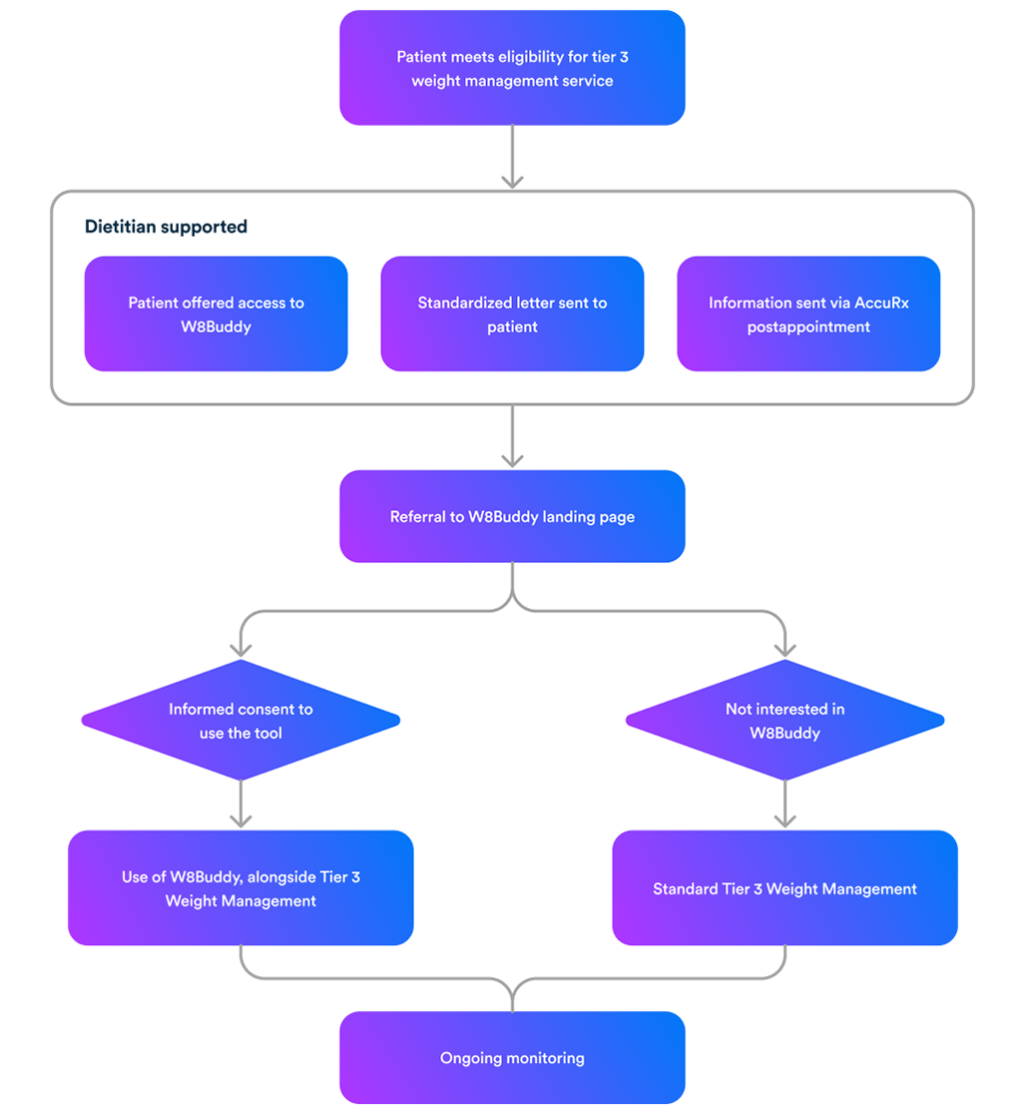
Patient journey through the service.

Following the incorporation of W8Buddy into the standard care, from September 2022, all patients accessing tier 3 WMS at the UHCW were offered access to the digital support tool. The referral criteria for tier 3 WMS were BMI over 40 kg/m^2^ or over 35 kg/m^2^ with medical conditions (eg, diabetes, hypertension, or polycystic ovary syndrome [PCOS]). Patients were informed about W8Buddy while waiting for their first dietetic appointment, following a first review by a dietetics practitioner who acted as a digital coach and explained what their journey in the service would be like. This was followed by a standardized letter or a message with information about W8Buddy via a safe messaging system (AccuRx), after their first dietetic or psychology appointment. This provided instructions on how to access the landing page [19] with detailed information about W8Buddy. Interested patients then proceeded to provide informed consent to be able to use the digital tool. Patients had an option to provide a second consent to use their anonymized data for research purposes, however, this was not mandatory to access the digital tool. Due to a long backlog of patients waiting for a dietetic appointment due to the COVID-19 pandemic, the time frame when W8Buddy was offered differed between patients, depending on the wait time for a first dietetic appointment. For the purposes of this study, patients who chose to use W8Buddy are referred to as W8Buddy users, whereas those who did not are referred to as control group participants. Figure 2 provides information on the patient journey through the service.

### Research Design

This project was registered as a service evaluation with a local hospital’s research and development department. Aligned with their clinical care, W8Buddy users did not receive any payment for engagement with W8Buddy. All patients received usual care regardless of their choice to use W8Buddy, as this was only an additional support tool in the service, and it did not replace the standard face-to-face care. There was no cost to patients or the NHS for accessing W8Buddy.

### Data Collection

Data were collected by 3 clinical members of this study’s team working in the specialist obesity service from October 2022, using the UHCW hospital record system. Members of the clinical team had access to the clinician dashboard of the digital tool, where they could monitor patients’ engagement with the app. Uptake was assessed by the proportion of patients who activated the digital tool after receiving information about it. Engagement was assessed by the proportion of patients who were using the digital tool at monthly intervals, with engagement defined as opening the web or mobile platform and inputting an item of data in the prior 14 days. Information about who was provided access to W8Buddy was constantly updated on the hospital database to provide up-to-date information about the activation rate.

Clinical outcome measures included information retrieved from standard clinical records, such as data on age, gender, ethnicity, comorbidities (presence of T2DM, PCOS, etc), body weight, and HbA_1c_. Data on body weight were collected at the time when participants first started in the WMS, when information was provided to them about W8Buddy, and then at subsequent follow-up times in the service. It was not possible to collect outcomes at the same time points for all study participants, but rather when they were available on the hospital system, based on the timings of clinical appointments.

Optional validated surveys (collected in-app) included the Satisfaction With Life Scale (SWLS), EQ-5D-5L, Patient Health Questionnaire (PHQ-8), and Karolinska Sleepiness Scale. The data from psychological surveys were provided to members of the clinical team as an anonymous aggregate dataset.

Participant engagement and ongoing cocreation was an important part of this study following its launch and several engagement events were held between April and July 2023 to identify ways to improve engagement with W8Buddy and encourage ongoing cocreation with users. Engagement workshops were recorded following participants’ verbal consent and key themes were summarized following each event.

### Statistical Analyses

Baseline data, describing the characteristics of this study’s populations (eg, age or gender), were summarized using means and SDs (for continuous data), and counts and percentages (for categorical data) for both the W8Buddy user and control groups. Comparisons between populations (W8Buddy vs control) were made using (independent samples) *t* tests and Fisher exact tests, for continuous and categorical outcomes, respectively. Multiple linear regression analysis was used to explore associations between a participant’s weight and their time spent in this study (months), for the W8Buddy and control populations. A participant’s current weight was related to their initial study (starting) weight; therefore, the latter was included as a covariate in all linear regression models, along with gender, which was expected to be strongly associated with weight loss. The effects of W8Buddy use and participant baseline data on weight were explored by including each of the variables in linear regression models and testing for model improvement using likelihood ratio tests. A simple linear (straight line) term was used to crudely model the relationship between time in this study and weight loss, mainly as a means to investigate whether there was any evidence in the data to support such a significant relationship. In reality, a linear relationship is unrealistic, but a plateauing effect for weight loss with time is much more likely. However, there were insufficient numbers of participants and follow-up duration to explore relationships more complex than linear. All analyses were on a complete case basis, and significance was assessed at the 5% level. All analyses were undertaken in R (R Foundation) [20].

### Ethical Considerations

No separate ethical approval was necessary as the use of W8Buddy was part of standard clinical care. For NHS service evaluation, ethical approval is not needed. The study reference number was SE0332. Users of W8Buddy app provided informed consent prior to the app use. For the routinely collected health data, no informed consent was needed. There was no compensation for this study as it was a service evaluation and involved observation of routinely delivered care.

## Results

### Descriptive Characteristics

Complete weight datasets were analyzed at the end of September 2023 (capturing 12 months of W8Buddy use). Participants’ complete weight data were available from 412 individuals accessing tier 3 WMS (220 W8Buddy users and 192 control nonusers). Due to the ongoing nature of data collection, the time in the service differed between participants depending on their study start date. Participants’ baseline characteristics are summarized in Table 1.

**Table 1:** Baseline characteristics of study participants at the time of either the start of W8Buddy use or continuation of WMS^a^ (control).

Baseline characteristic			Control (n=192)	W8Buddy (n=220)		Total (N=412)		*P* value^b^	
**Gender, n (%)**									.11
	Female	149 (77.6)		185 (84.1)	334 (81.1)				
	Male	43 (22.4)		35 (15.9)	78 (18.9)				
Age (years), mean (SD)			46.1 (13)	43.4 (10.8)		44.7 (11.9)		.02	
**Ethnicity, n (%)**									<.001
	African	0 (0)		3 (1.4)	3 (0.7)				
	Asian	1 (0.5)		0 (0)	1 (0.2)				
	Bangladeshi	0 (0)		2 (0.9)	2 (0.5)				
	Black	9 (4.7)		5 (2.3)	14 (3.4)				
	White British	128 (66.7)		159 (72.3)	287 (69.7)				
	Chinese	1 (0.5)		0 (0)	1 (0.2)				
	Indian	3 (1.6)		2 (0.9)	5 (1.2)				
	Other	8 (4.2)		27 (12.3)	35 (8.5)				
	Pakistani	2 (1)		1 (0.5)	3 (0.7)				
	Unknown	39 (20.3)		21 (9.5)	60 (14.6)				
	White other	1 (0.5)		0 (0)	1 (0.2)				
T2DM^c^, n (%)			40 (20.8)	45 (20.5)		85 (20.6)		.91	
OSA^d^, n (%)			41 (21.4)	43 (19.5)		84 (20.4)		.71	
Hypertension, n (%)			51 (26.6)	66 (30)		117 (28.4)		.45	
PCOS^e^, n (%)			23 (12)	44 (20)		67 (16.3)		.03	
T1DM^f^, n (%)			1 (0.5)	0 (0)		1 (0.2)		.466	
High cholesterol, n (%)			19 (9.9)	27 (12.3)		46 (11.2)		.54	
Heart failure, n (%)			2 (1)	5 (2.3)		7 (1.7)		.46	
Osteoarthritis, n (%)			33 (17.2)	26 (11.8)		59 (14.3)		.12	
Starting weight (kg), mean (SD)			130.2 (29.4)	134 (23.2)		132.3 (26.2)		.14	
Starting BMI (kg/m^2^), mean (SD)			46.8 (7.8)	49.6 (12.3)		48.2 (10.5)		.008	
Months in study, mean (SD)			6.7 (3.2)	4.1 (2.4)		5.3 (3.1)		<.001	

^a^WMS: weight management service.

^b^*P* values from *t* test (continuous data) or Fisher exact test (categorical data).

^c^T2DM: type 2 diabetes mellitus.

^d^OSA: obstructive sleep apnea.

^e^PCOS: polycystic ovary syndrome.

^f^T1DM: type 1 diabetes mellitus.

### Activation and Retention

Activation was assessed in June and December 2023. A total of 783 patients were informed about W8Buddy by the end of June 2023. A total of 220 patients activated the tool and started using it (220/783, 28.1%) by end of June 2023. By the end of December 2023 there were 1170 patients informed about W8Buddy and 392 (33.5%) activated it.

In December 2023, a total of 326 (83.1%) participants actively engaged with the platform within the previous 28 days, which required logging in and inputting data. Just over half (200/392, 51%) completed the digital program (defined as completing ≥12 of the 16 education modules available).

### Weight Changes

**Figure 3:**
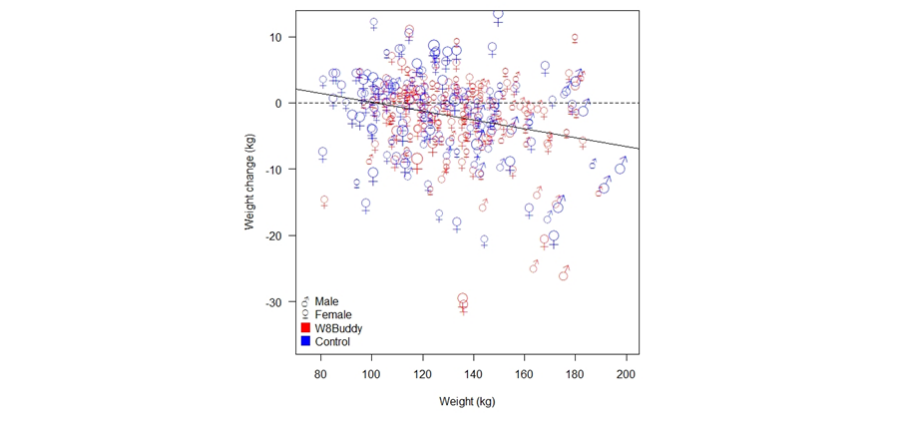
Participant weight change versus initial (starting) study weight, annotated by gender and group, and symbols sized to show the number of months in this study (a larger symbol indicates a larger study time), and fitted linear regression model (—).

Figure 3 shows the weight change for each study participant plotted against their initial weight.

The plot symbols in Figure 3 show participant gender and are scaled to show the number of months in this study (5 categories given by 0-3, 3-6, 6-9, 9-12, and 12-15 months). The line shows the linear regression fit, which was statistically significant (*P*<.001), indicating that there was greater weight loss for participants with a larger starting weight.

**Figure 4:**
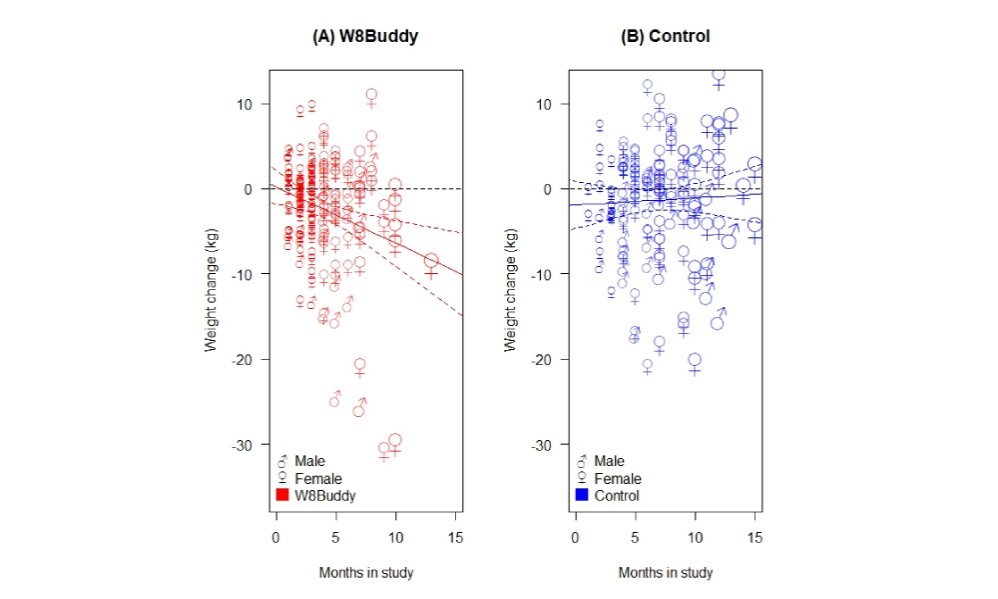
Participant weight change versus months in this study, annotated by gender and symbols sized to show the number of months in this study (a larger symbol indicates a larger study time), and fitted linear regression models (—) for (A) the W8Buddy user group and (B) the control group.

There was also strong evidence that the weight change was related to the number of months in this study for the W8Buddy group, but not for the control group (Figure 4).

The lines in Figures 4A and 4B show fitted linear regressions, which indicate there was a statistically significant relationship between weight change and months in this study for the W8Buddy group (*P*=.05), but not for the control group (*P*=.65).

Fitting a linear regression model for weight, including initial (starting) weight, gender, months in study, and a treatment factor (W8Buddy or control), gives the estimated regression coefficients in Table 2.

**Table 2:** Estimated coefficients for linear regression model for current participant weight, including initial weight, gender, months in this study, and treatment (W8Buddy or control group), and interaction between the latter 2 terms.

Coefficient	Estimate	95% CI	*P* value
Initial (starting) weight	0.95	0.92 to 0.99	<.001
Months in study	0.07	–0.26 to 0.41	.67
Gender: male	–2.82	–4.9 to –0.75	.008
Treat: W8Buddy	2.27	–0.92 to 5.46	.16
Months in study: W8Buddy	–0.74	–1.28 to –0.21	.007

Table 2 shows that the current weight of individual participants was (on average) 95% of the initial (starting) weight and that the male participants were (on average) 2.82 kg lighter than female participants. The highly statistically significant interaction term between study time and treatment (months in study: W8Buddy; *P*=.007) indicates that the time in this study was an important predictor of weight for those in the W8Buddy group only (ie, in the control and the number of months in this study was unimportant). In the W8Buddy group, there was a significant weight loss of –0.744 kg/month. The longer an individual stayed in this study and used W8Buddy, the more weight was lost.

The linear regression model including the treatment effect term (W8Buddy and control groups) and the interaction between months in this study and treatment was very strongly significantly better than the (base) model not including these terms (*P*=.005). Using the model to predict weight changes for both groups shows that, for instance, for a male with a starting weight of 130 kg, for 12 months, in the W8Buddy group would result in a weight change to 120 kg (a 7.7% change), and in the control group a change to 127 kg (a 2.3% change). Emphasizing the strength of the model interaction term between study time and weight change for the W8Buddy group.

### Changes in HbA_1c_

Out of 220 users, 45 had a diagnosis of T2DM (prevalence n=45, 20.5%). Paired data on HbA_1c_ were available from 27 users, and the mean difference between the baseline and follow-up HbA_1c_ was 8.9 months. There was a statistically significant reduction in HbA_1c_ from 59.8 mmol/mol to 51.2 mmol/mol (*P*=.02).

Out of 192 nonusers, 40 nonusers had a diagnosis of T2DM (prevalence of n=40, 20.8%). Paired data on HbA_1c_ were available from 32 nonusers, and the mean difference between the baseline and follow-up HbA_1c_ was 10.7 months. There was a reduction in HbA_1c_ from 62.5 mmol/mol to 60 mmol/mol, but it did not reach statistical significance (*P*=.24). Comparing the difference in HbA_1c_ between the W8Buddy and control group, there was a numerical difference of 6.1 mmol/mol, however, this was not statistically significant (*P*=.14). This is summarized in Table 3.

**Table 3:** Differences in HbA_1c_^a^.

Group	Baseline HbA_1c_ in mmol/mol (SD)	Follow-up HbA_1c_ in mmol/mol (SD)	Mean difference in HbA_1c_ mmol/mol (SD)	Months for follow-up HbA_1c_ (mean)	*P* value
W8Buddy (n=27)	59.8 (16.3)	51.2 (9.9)	8.7 (17.8)	8.9	.02^b^
Control (n=32)	62.5 (17.4)	60 (16.4)	2.5 (12.1)	10.7	.24^b^
W8Buddy vs control	N/A^c^	N/A	6.1 (4)	N/A	.14^d^

^a^HbA_1c_: hemoglobin A_1c_.

^b^*P* values from paired *t* test.

^c^N/A: not applicable.

^d^*P* values from an independent *t* test.

### Psychological Measures

Psychological outcome measures were collected via the digital tool as an optional survey. As a result, only a proportion of the user sample (W8Buddy group) completed the validated surveys. The results are summarized in Table 4.

**Table 4:** Differences in psychological outcomes among the W8Buddy group.

Psychological survey	Baseline (SD)	Follow-up (SD)	Mean difference (SD)	Length of follow-up	*P* value^a^
SWLS^b^ (n=81)	19.5 (4.8)	23 (3.7)	3.5 (2.7)	111.4	<.001
PHQ-8^c^ (n=108)	15.2 (2.6)	15.4 (4)	0.2 (2.2)	200	.41
KSS^d^ (n=100)	3.9 (1.6)	3.2 (1.5)	–0.7 (0.8)	112.7	<.001
EQ-5D VAS^e^ (n=144)	60.4 (19.6)	72 (19)	11.6 (4.8)	112.1	<.001
EQ-5D HIS^f^ (n=127)	0.76 (0.2)	0.77 (0.2)	0.01 (0.3)	112.1	.58

^a^*P* values from paired *t* test.

^b^SWLS: Satisfaction With Life Scale.

^c^PHQ-8: Patient Health Questionnaire.

^d^KSS: Karolinska Sleepiness Scale.

^e^VAS: visual analog scale.

^f^HIS: health index score.

### About SWLS

Paired data were available from 81 users. The mean difference between baseline and a follow-up survey completion was 111.4 days. There was a statistically significant improvement in satisfaction with life from 19.5 to 23 (*P*<.001).

### About PHQ-8

Paired data from PHQ-8 were available from 108 users. The mean difference between the competition of baseline and follow-up surveys was 200 days. There was no difference between baseline and follow-up scores (from 15.2 to 15.4, *P*=.41).

### Karolinska Sleepiness Scale

Paired data were available from 100 users. The mean difference between baseline and a follow-up survey completion was 112.7 days. There was a statistically significant reduction in the Karolinska Sleepiness Scale from 3.9 to 3.2 (*P*<.001).

### Quality of Life Score (EQ-5D-5L)

Paired data were available from 144 users. The mean difference between baseline and a follow-up survey completion was 112.1 days. There was a statistically significant improvement in the visual analog scale value of quality of life from 60.4 to 72 (*P*<.001). There was no change in the health index score from 0.76 to 0.77 (*P*=.59).

### Patient and Public Involvement

In summer 2023, a total of 4 PPI workshops were held on MS Teams (Microsoft Corp). All participants [15] were users of W8Buddy and gave verbal consent to take part in the online workshop and were happy for the themes from the workshops to be summarized. These workshops aimed to identify how to improve the content and the process of offering W8Buddy to patients. Most patients were in tier 3 WMS for 6-12 months before they were offered W8Buddy. The themes are summarized in Textbox 1.

These workshops provided some understanding of what can be improved to encourage higher uptake from patients. This included simplification of the welcome screen and providing guidance on how to work with the digital tool, offering W8Buddy at the very beginning of a patient journey in tier 3 WMS, and linking it with general practitioner referral for users to open before the first appointment in the service. This led to the creation of short videos that guide users on how to work through the rich content and how to ask for help if unsure where to begin. Participants also commented on the importance of NHS staff awareness on how to help with onboarding. At UHCW tier 3 WMS, we now have 2 dietetic practitioners who support patients with using W8Buddy.

**Themes from patient and public engagement workshops.**

**Why did you start using it?**

- Recommended by health care professionals
- Self-recording and tracking
- To have another source of support
- To minimize the number of health-related apps, as W8Buddy had all in one

**What do you think of it, and how did you use it?**

- Dietetic component—recipes as a resource
- Sleep
- Activity component-monitoring and short exercise snacks
- Overall support—It helped to know that there was some support between appointments
- Useful to see familiar faces (health care professionals in the videos)

**How can we improve it?**

- Link W8Buddy to the referral process to tier 3 from the general practitioner
- Include price on each recipe
- Offer exercise advice for people with severe mobility issues and paralysis
- Have a troubleshooting page (eg, how to integrate it with other health apps)
- Have diet contents for special diseases, for example, ulcerative colitis
- Streamline the info and simplify the welcome screen
- Gamification of health behaviors
- Move onboarding meetings after 5 PM

### Adverse Events

No adverse events were reported throughout this study.

## Discussion

### Principal Findings

Implementation of the W8Buddy tool within the tier 3 WMS at UHCW demonstrates promising results in several key areas. This is the first study of its kind, incorporating a specifically designed digital tool into the NHS specialist weight management and offering patients the digital tool for free.

Acceptance data indicates that a third of those who were informed about the tool decided to activate and use it (n=392, 33.5%), suggesting that a significant number of patients are open to using digital tools as a resource in managing their weight. However, it demonstrates the need for further strategies to enhance engagement and uptake, possibly through more personalized approaches.

Concerning clinical outcomes, there was a statistically significant difference between the weight loss achieved by patients engaging with W8Buddy as part of usual WMS compared to the traditional WMS alone. Even though the impact on weight was modest (0.744 kg improved weight loss per month), over time, this could lead to a clinically significant difference for patients and their overall health. The degree of weight loss correlated with the duration of usage of the digital tool, implying that app engagement resulted in sustained lifestyle changes and steady weight loss that was durable at least over the 4-month duration of this study.

Outcomes from traditionally delivered tier 3 WMSs are mixed, with 5% weight loss in 50% of patients over 6-12 months, with retention rates below 55% and no long-term data on sustainability of weight loss [21]. A systematic review of tier 3 specialist WMS and prebariatric multicomponent weight management programs for adults with obesity living in the United Kingdom found that at 6 months, 11 (58%) studies reported changes in BMI or weight (kg) or both—with an average weight change of 5.71 kg at 6 months reported by the included studies [22]. A study in Birmingham and Solihull found that a tier 3 WMS received 421 referrals with an average baseline weight of 128.5 kg and 32% T2DM prevalence, comparable to our study. There was a 65.6% dropout rate, with 110 patients completing at 12 months. The results show 61 (14%) participants lost a median weight of 4.08 kg, with 45 (10%) participants gaining weight [23].

Evidence for the effectiveness of digital tools (without additional weight loss medication) for weight management outside of NHS services varies from 13% weight lost at 4 months using an entirely remote program with meal replacements [24] to 7.6% weight loss at 12 months in a digital intervention with one-to-one coaching [25]. A study of ImpulsePal, a digitally delivered weight management intervention targeted at impulsive eaters, found an average weight loss of 1.03 kg at 3 months [26]. A recent study found that 24.9% of users of digital weight management interventions recorded outcomes at 2 years, with 18.1% of users achieving 5% weight loss at 2 years [14]. However, none of these studies assessed a digital tool offered as an integrated part of the clinical pathway in the NHS tier 3 WMS.

The data demonstrates a statistically significant reduction in HbA_1c_ levels over an average period of 8.9 months, which is a positive indicator of improved glycemic control among users with T2DM. This finding underscores the potential of digital tools such as W8Buddy in supporting individuals in managing chronic comorbidities more effectively. However, it is important to point out that even though nonusers did not experience any statistically significant changes to their HbA_1c_ between the 2 time points, the difference in HbA_1c_ reduction over time between users and nonusers did not reach statistical significance. Importantly, no information regarding glycemic therapy was collected as part of this study, and hence it is not possible to discern the extent to which the W8Buddy intervention contributed toward HbA_1c_ improvement, as compared to other antiglycemic therapies.

The psychological measures collected through the tool further substantiate its potential benefits. Users reported a significant improvement in life satisfaction, as evidenced by the increase in scores on the SWLS. This is supported by a significant reduction in sleepiness as measured by the Karolinska Sleepiness Scale, which might imply an improved sleep quality or pattern, potentially contributing to better overall health and well-being. There was no change in PHQ-8 scores, indicating no change in depressive symptoms among participants. This is similar to findings of a recent digital weight loss study that found no difference in PHQ-9 scores among completers [27].

Quality of life, as measured by the EQ-5D-5L scale, showed significant improvements in the visual analog scale value, which saw a substantial increase. Although the improvement in the health index score was not statistically significant, it showed a very slight positive trend, hinting at potential benefits that might become more apparent with a larger sample size or over a longer period.

The advent of precision digital technologies in the health care sector marks a significant stride toward fostering personalized patient care, integrating with traditional health care, be it in-person or remote consultations. Our initiative of developing W8Buddy is a testament to this progressive shift, facilitating a comprehensive and nuanced approach to weight management that encompasses various facets, including social, dietary, and psychological dimensions [28]. These advanced digital platforms are adept at adapting based on individual patient data, thereby paving the way for more targeted and effective interventions. The feature of remote self-monitoring and self-reporting of body weight not only empowers patients to be active participants in their health care journey but also serves as a valuable tool for health care professionals to streamline resource allocation, enhancing the efficiency of health care delivery. Additionally, a meta-analysis showed that adding self-weighing to a behavioral weight loss program may improve weight loss [29], which could have contributed to the observed weight changes.

Furthermore, these digital interventions have the potential to significantly enrich the patient experience within the realm of WMSs. They not only foster successful weight loss outcomes but also act as a conduit for improved communication between patients and health care professionals, fostering a collaborative and supportive health care environment. Precision digital technologies stand as a beacon of innovation in health care, promising a future where care is not only more personalized but also more efficient and collaborative, fostering better outcomes and improved patient experiences.

Our study has several limitations. As this study assessed the outcomes of the W8Buddy tool used as part of the existing tier 3 service, we did not collect information on medication changes that future studies should capture. Change in glycemic therapy could be a confounder, given the effects of SGLT2 (sodium glucose-like cotransporter inhibitor) inhibitors and glucagon-like peptide-1 analogues on body weight. However, it is reasonable to assume that changes in medication occurred similarly across the patients accessing the service.

Additionally, given the observational nature of this study, confounders (such as readiness to change) could not be removed, and there was likely a difference between those patients who chose to use W8Buddy and those who did not. However, characteristics of users and nonusers were similar. Data collection was carried out based on clinical appointments, which varied in time, with many occasions where weight was not recorded during the clinical appointment. This impacted the length of follow-up data available for weight changes among users (only 4.1 months) and also explained why some parameters (such as HbA_1c_, collected via blood test) were collected more reliably and thus available for analysis at a later time point. It is therefore crucial to obtain longer-term data to understand whether this weight loss trajectory continues over time or whether it plateaus, given the mean follow-up time for patients accessing W8Buddy was 4.1 months.

A strength of this study is that data for people who were offered access to the digital tool but chose not to accept it or did not complete the sign-up were tracked and followed up. This enabled comparison to users. An additional strength of this study is the significant patient contribution in the form of ongoing patient workshops and ongoing multidisciplinary team input.

The level of patient engagement with the app during the follow-up phase was notably high. This heightened engagement could be attributed to several factors: first, the app was provided to patients at no financial cost, a practice that aligns with the UK’s health care policy as part of their tier 3 weight management care. This not only mirrors the real-world scenario of this study but also potentially fosters higher engagement levels with the app.

### Conclusion

The integration of the W8Buddy tool as part of the tier 3 WMS at UHCW has showcased promising advancements in the realm of digital health care, particularly in fostering personalized patient care that integrates seamlessly with traditional tier 3 weight management. The tool seems to foster positive changes in lifestyle and health management, which are reflected in the improvements in glycemic control, mental well-being, sleep quality, and perceived quality of life.

It would be beneficial to continue monitoring the tool’s cost-effectiveness and exploring strategies to enhance user engagement further. Additionally, future research could delve deeper into understanding the specific elements of the tool that are most beneficial to users and how it can be further optimized to support individuals in their weight management journey.

Data thus far suggests that digital tools such as W8Buddy can play a pivotal role in modern health care delivery, offering a supportive platform that complements traditional health care services and promotes patient empowerment and self-management.

## Abbreviations

HbA_1c_: hemoglobin A_1c_
NHS: National Health Service
ORCHA: Organisation for the Review of Care and Health Applications
PCOS: polycystic ovary syndrome
PHE: Public Health England
PHQ: Patient Health Questionnaire
SGLT2: sodium glucose-like cotransporter inhibitor
SWLS: Satisfaction With Life Scale
T2DM: type 2 diabetes mellitus
UHCW: University Hospitals Coventry and Warwickshire
WMS: weight management service
